# Exploring comorbid use of marijuana, tobacco, and alcohol among 14 to 15-year-olds: findings from a national survey on adolescent substance use

**DOI:** 10.1186/s12889-015-1585-9

**Published:** 2015-03-10

**Authors:** Joanna White, Darren Walton, Natalie Walker

**Affiliations:** Health Promotion Agency, Wellington, New Zealand; University of Canterbury, Christchurch, New Zealand; National Institute for Health Innovation, University of Auckland, Auckland, New Zealand

**Keywords:** Adolescence, Youth, Comorbid substance use, Marijuana, Tobacco, Alcohol

## Abstract

**Background:**

Understanding the patterns of comorbid substance use, particularly among adolescents, is necessary to address resulting harm. This study investigated the prevalence of comorbid use of marijuana, tobacco and binge drinking among 14 to 15-year-olds. The study also examined the relationship between comorbid substance use and behaviour frequency and explored common underlying risk factors for comorbid substance use.

**Methods:**

A nationally representative sample of 3,017 New Zealand Year 10 students completed self-report measures of marijuana use, tobacco use, binge drinking and socio-demographic characteristics in the 2012 Youth Insights Survey (YIS). Weighted population estimates were calculated. Ordinal logistic regression models were constructed to a) investigate the relationship between comorbidity and substance use behaviour frequency, and b) profile those with the greatest degree of comorbid substance use.

**Results:**

In the past month, one-in-twenty (4.7%) students had engaged in all three substance use behaviours, 5.8% in two, and 11.9% in one. Around half of adolescents who had engaged in one had also engaged in another, with three-quarters of tobacco-users also using marijuana and/or binge drinking. Respondents who reported a greater degree of comorbidity were likely to engage in substance use behaviour more frequently. Comorbid substance use was significantly predicted by gender, ethnicity, school decile status, past week income, social connectedness, and parental monitoring and rule enforcement.

**Conclusions:**

The results identify a core group of adolescents sharing common characteristics who frequently engage in comorbid substance use behaviours. More sophisticated and wider interventions addressing multiple substances are required, especially for marijuana and tobacco use.

## Background

The harm associated with the use of marijuana, tobacco and alcohol in adolescence is substantial. For instance, marijuana appears to disrupt brain development and adolescent users of marijuana have a greater risk than adult users of developing hallucinations and psychosis [1]. Earlier onset of smoking tobacco has been shown to increase nicotine dependence and lead to heavier use and greater likelihood of continued smoking [2]. Binge drinking (i.e., consuming relatively large amounts of alcohol in a single occasion) in adolescence is associated with health risk behaviours such as interpersonal violence, risky sexual behaviour, attempted suicide, travelling in a car with a driver who had been drinking, as well as poor school performance [3].

In New Zealand, a sixth of school exclusions for students under the age of 16, where the student cannot return to the school and must enrol elsewhere, are due to alcohol, tobacco, or drug use [4]. In 2012, 8% of New Zealand adolescents aged 14 to 15 years were regular smokers (smoking daily, weekly, or monthly) [5], while 13% of 13 to 17-year-olds were current marijuana users and 23% had engaged in binge drinking in the past month [6].

The use of marijuana, tobacco and alcohol tends to cluster in adolescent populations, with the use of one of these substances increasing the likelihood of the use of others [7-9]. Comorbid use of marijuana, tobacco and alcohol, where all three of these substances are used concurrently or within a short time period, is of particular concern because comorbid substance use in adolescence has been related to heavier patterns of consumption in adulthood compared with singular or dual use [10-12]. Similarly, a cross-sectional evaluation of drug use across 17 countries, including the United States and New Zealand, found that a greater number of substances used and earlier onset of use predicted later substance dependence [13]. Further evidence for the resulting harm associated with comorbid substance use in adolescence comes from studies which show that it is related to frequency of substance use. Adolescents who engage in more frequent marijuana use, tobacco use and binge drinking are at greater risk of negative outcomes such as poor mental health and school performance [12], addiction [2], and engagement in other health risk behaviours [3].

The extent to which comorbid substance use in adolescence is driven by one substance acting as a ‘gateway’ for another, or through an underlying vulnerability to substance use in general, is unclear. The Gateway Theory posits that substance use initiation follows a sequential pattern, where the use of licit substances such as tobacco or alcohol lead to future risk of using marijuana, which in turn leads to future risk of using other illicit substances [14]. A ‘reverse’ gateway effect has also been noted, where frequent marijuana use predicts future tobacco use and dependence [15]. Evidence for a common underlying vulnerability to substance use comes from associations such as those found between externalising behaviour problems and adolescent substance use [16-18]. Further, studies have found that relationships between the use of marijuana, tobacco and alcohol in adolescence could be predicted by risk factors such as parental and peer substance use [19], greater amount of weekly spending money [20] and permissive parental rules on alcohol [21].

The current study examined the patterns of comorbid marijuana use, tobacco use and binge drinking among a nationally representative sample of 14 to 15-year-old students in New Zealand. The study had three aims:

1. to identify the prevalence of comorbid substance use among New Zealand adolescents
2. to examine the relationship between the frequency of marijuana use, tobacco use and binge drinking and the comorbid combination of these behaviours
3. to profile those engaging in comorbid substance use in order to determine underlying risk factors and a common vulnerability to adolescent substance use.

## Methods

Data were sourced from the 2012 New Zealand Youth Insights Survey (YIS). The YIS is a biennial survey that monitors risk and protective factors that relate to smoking uptake among adolescents. Written consent to participate was given by school principals and deputy school principals on behalf of their students. Given the anonymity of the survey and the minimal risk of harm to students it was not considered necessary to seek active consent from parents [22], although parents were advised through school newsletters that their child’s school had been invited to take part, the details of the survey, and that their child would decide whether or not to participate.

Participants completed the questionnaire anonymously in a classroom setting under supervision of their teacher and a trained fieldworker. Participants were informed that their responses would remain anonymous and that participation was voluntary, and were asked to tick a box on the front of the questionnaire to indicate their consent. Participants did not put their name on the questionnaire and neither teachers nor parents were permitted to look at the questionnaires after completion. The questionnaire took on average 45 minutes to complete. Participating schools were not named or identified in any way in any publications or to the media. Ethical approval for the YIS was granted by the Ministry of Health’s multi-regional ethics committee in 2007.

### Participants and sampling procedure

The YIS uses a two-stage cluster sample design to obtain a nationally representative sample of New Zealand Year 10 students (predominantly 14 and 15-year-olds). A sample of 186 schools was randomly drawn from the list of all eligible schools with Year 10 students in New Zealand. Probability of school selection was proportional to roll size. One Year 10 class in each selected school was then randomly drawn from a list of all mutually exclusive Year 10 classes. Each Year 10 student had only one chance to participate and an equal opportunity of selection. All students in selected classes were invited to participate. Further detail on the YIS methodology is described in a methodology report [23].

### Outcome measures

The YIS questions used for the outcome measures were aligned with other similar youth surveys [6,24]. Following the recommendation of previous research [9] this study considers substance use in the past month rather than lifetime use. Marijuana use was measured by the question, “During the past 30 days (one month), how often did you smoke marijuana (pot, grass, weed, cannabis)?” Past-month marijuana smokers were defined as students who reported they had smoked marijuana at least once in the past month. Tobacco use was measured by the question, “During the past 30 days (one month), on how many days did you smoke cigarettes?” Past-month tobacco smokers were defined as students who reported they had smoked cigarettes on at least one day in the past month. Finally, binge drinking was measured by the question, “During the past 30 days (one month), about how often did you have 5 or more alcohol drinks in one session? (count one drink as one small glass of wine, one can or stubbie, or one ready-made alcohol drink, e.g. rum and Coke or one nip of spirits)”. Past month binge drinkers were defined as students who reported they had done this on at least one occasion in the past month. Five or more drinks in one session is the adult male limit for risk of injury recommended in low-risk alcohol drinking advice [25]. This level of drinking is therefore a conservative estimate of alcohol-related harm among this survey population.

Demographic variables recorded included gender, prioritised ethnic identification (Māori or non-Māori), and school decile status (low, mid or high) as a proxy for socio-economic status. Prioritised ethnic identification allocates individuals who identify with more than one ethnic group to a single ethnic group based on whether or not they identified with Māori ethnicity [26]. School decile status was collapsed into ‘low’ (deciles 1 to 4, most deprived), ‘mid’ (deciles 5 to 7), and ‘high’ (deciles 8 to 10, least deprived).

Past week income was assessed by the question, “In the past 7 days (one week), how much money did you get or earn ($ per week)?” Responses were collapsed into four categories (“none”, “$1 to $15”, “$16 to $30”, and “more than $30”^a^) with a roughly even proportion of respondents in each.

Social connectedness was measured by combining all eight items relating to family, peer, and school connectedness (Cronbach’s alpha = 0.78), where respondents were asked the degree to which they agreed with each statement on a five-point scale ranging from “strongly agree” to “strongly disagree”. The eight items were: “I like to spend free time with my family/whānau”; “We can easily think of things to do together as a family/whānau”; “My family/whānau ask each other for help”; “I can trust my friends with personal problems”; “My friends understand and accept me for who I am”; “I feel I am treated with as much respect as other students at school/kura”; “I like going to my school/kura”; and, “I feel proud to say what school/kura I go to”. Scores were collapsed into ‘low’, ‘medium’ and ‘high’ social connectedness based around the three quartile distribution for the sample.

Parental monitoring of expenditure, monitoring of whereabouts and rule enforcement were determined by agreement with three statements: “My parents or caregivers generally know what I spend my pocket money on”; “My parents or caregivers often have no idea of where I am, when I am away from my home” (reverse scored); and, “If I break any important rules that my parents or caregivers have set I always get into trouble”. Responses for each statement were grouped into “agree” and “disagree or don’t know”.

### Analysis

The sample was weighted to adjust for non-response, selection probability and to match the sample’s gender and ethnicity breakdown with the total for all New Zealand Year 10 students. Weighted population estimates were calculated for substance use behaviour prevalence and the outcome measures. Ninety-five percent confidence intervals (95% CI) were calculated using jack-knife variance estimation. Analyses were carried out using Stata IC version 12.0, and were restricted to students aged 14 to 15 years [27].

To determine whether those engaging in a greater number of substance use behaviours were likely to undertake each more frequently, three ordinal logistic regression models [28] were constructed with the dependent measure being frequency of substance use behaviour and the independent measure being whether one, two, or all three behaviours were present^b^. To determine the profile of those with the greatest degree of comorbidity, all socio-demographic predictors were entered into another ordinal logistic regression model with the dependent measure being whether one, two, or all three behaviours were present.

## Results

Of the 186 schools selected from all New Zealand schools, 147 agreed to participate, giving a 77% school-level response rate. Completed questionnaires were received from 3,143 students, which gave a student-level response rate of 82% and an overall response rate of 65%. The analysis sample (n = 3,017) comprised 49.0% female (n = 1,435) and 22.9% people of Māori ethnicity (n = 681). In the past month, 10.1% had smoked marijuana, 11.4% had smoked tobacco, and 16.8% had engaged in binge drinking. Table 1 describes the weighted proportion frequency and prevalence estimates of the substance use behaviours in more detail. Table 2 presents the weighted proportion frequency estimates of the behaviours by socio-demographic characteristics.

**Table 1:** **Prevalence and frequency of substance use behaviours in the past month**

	**n**	**Weighted % (95% CI)**
Prevalence of marijuana use in past month		
Smoked marijuana in past month	290	10.1 (8.7-11.5)
Did not smoke marijuana in past month	2680	89.9 (88.5-91.3)
Frequency of marijuana use in past month		
Never at all	2426	81.0 (78.8-83.1)
In the past but not in the past 30 days	254	8.9 (7.5-10.3)
Once	62	2.2 (1.6-2.7)
Two or three times	111	3.8 (3.1-4.6)
Once a week	32	1.1 (0.7-1.6)
Several times a week	37	1.3 (0.8-1.8)
Every day	17	0.5 (0.3-0.8)
Several times a day	31	1.1 (0.7-1.6)
Prevalence of tobacco use in past month		
Smoked cigarettes on at least one day in past month	335	11.4 (9.8-12.9)
Did not smoke cigarettes on any days in past month	2658	88.6 (87.1-90.2)
Frequency of tobacco use in past month		
0 days	2658	88.6 (87.1-90.2)
1 to 2 days	123	4.1 (3.3-4.8)
3 to 5 days	46	1.6 (1.2-2.0)
6 to 9 days	19	0.6 (0.3-0.9)
10 to 19 days	43	1.5 (1.0-2.1)
20 to 29 days	27	0.9 (0.6-1.3)
All 30 days	77	2.6 (1.9-3.4)
Prevalence of binge drinking in past month		
Binge drinking in past month	472	16.8 (15.0-18.6)
No binge drinking in past month	2421	83.2 (81.4-85.0)
Frequency of binge drinking in past month		
Never at all	2025	69.4 (67.1-71.7)
In the past but not in the past 30 days	396	13.8 (12.3-15.2)
Once	169	6.0 (5.0-7.0)
Two or three times	184	6.6 (5.5-7.6)
Once a week	62	2.2 (1.5-2.8)
Several times a week	36	1.3 (0.9-1.8)
Most days	21	0.8 (0.3-1.2)

**Table 2:** **Past month substance use behaviour by socio-demographic characteristics**

	**Smoked marijuana**		**Smoked tobacco**		**Binge drinking**	
	**n**	**Weighted % (95% CI)**	**n**	**Weighted % (95% CI)**	**n**	**Weighted % (95% CI)**
Gender						
Female	142	10.1 (8.3-12.0)	186	13.4 (11.0-15.7)	233	17.4 (14.9-19.9)
Male	148	10.1 (8.3-11.8)	149	9.5 (7.8-11.2)	239	16.2 (13.8-18.6)
Ethnicity						
Māori	153	23.0 (18.7-27.4)	147	21.7 (17.8-25.6)	198	31.3 (27.0-35.6)
Non-Māori	137	6.3 (5.1-7.6)	188	8.4 (6.9-9.8)	274	12.6 (10.7-14.5)
School decile status						
Low	152	17.6 (13.7-21.5)	166	18.5 (14.6-22.4)	207	24.9 (20.8-29.0)
Mid	74	7.9 (5.9-9.9)	87	9.1 (6.5-11.7)	145	16.4 (12.8-20.1)
High	64	5.8 (4.2-7.4)	82	7.4 (5.6-9.2)	120	10.6 (8.3-13.0)
Past week income						
More than $30	104	17.3 (13.9-20.7)	106	17.3 (13.7-20.8)	166	28.3 (24.1-32.5)
$16 to $30	93	12.0 (9.6-14.4)	106	13.4 (11.0-15.9)	146	19.8 (16.7-23.0)
$1 to $15	45	5.7 (4.1-7.4)	65	8.0 (5.9-10.1)	77	9.6 (7.4-11.9)
None	45	6.7 (5.0-8.5)	56	8.2 (6.3-10.1)	79	11.9 (9.4-14.3)
Social connectedness						
Low	115	15.4 (12.3-18.4)	130	17.1 (14.0-20.1)	168	22.4 (19.2-25.5)
Medium	80	8.0 (6.0-10.0)	91	8.8 (6.7-10.9)	158	15.9 (13.0-18.8)
High	70	6.9 (5.0-8.8)	86	8.4 (6.5-10.3)	117	12.4 (9.9-14.9)
Parental monitoring of expenditure						
Disagree or don’t know	163	20.9 (17.6-24.2)	177	22.6 (19.1-26.0)	215	28.1 (24.6-31.7)
Agree	121	5.8 (4.7-6.9)	151	7.0 (5.7-8.3)	252	12.4 (10.6-14.2)
Parental monitoring of whereabouts						
Disagree or don’t know	119	22.3 (18.2-26.4)	147	27.0 (22.7-31.4)	170	32.9 (28.7-37.1)
Agree	166	7.1 (5.9-8.4)	182	7.6 (6.3-8.9)	299	13.0 (11.3-14.7)
Parental rule enforcement						
Disagree or don’t know	108	15.8 (12.2-19.3)	113	16.0 (12.7-19.2)	176	25.2 (21.9-28.5)
Agree	177	8.1 (6.8-9.4)	215	9.8 (8.2-11.3)	293	14.0 (12.1-15.9)

### Prevalence of comorbid substance use behaviours

Around one-in-five students (22.4%; 95% CI = 20.3-24.6) reported engaging in at least one of the substance use behaviours in the past month. One-in-twenty (4.7%; 95% CI = 3.7-5.7) had engaged in all three, 5.8% (95% CI = 4.9-6.7) in two, and 11.9% (95% CI = 10.4-13.4) in one only. The prevalence of each substance use behaviour configuration was: none = 77.6% (95% CI = 75.4-79.7); only binge drinking = 7.5% (95% CI = 6.3-8.7); only tobacco smoking = 2.6% (95% CI = 2.0-3.3); only marijuana smoking = 1.8% (95% CI = 1.1-2.4); binge drinking and tobacco smoking = 2.2% (95% CI = 1.6-2.8); binge drinking and marijuana smoking = 2.2% (95% CI = 1.6-2.8); tobacco and marijuana smoking = 1.4% (95% CI = 1.0-1.9); all three = 4.7% (95% CI = 3.7-5.7).

Of those who had engaged in one substance use behaviour in the past month, 46.9% had also engaged in at least one other. The weighted proportion estimates of co-occurring additional substance use behaviours are shown in Table 3. As shown, of those who had smoked tobacco in the past month, more than half (55.9%) had also smoked marijuana. A quarter (23.9%) had used tobacco in isolation, that is, without also using marijuana or binge drinking. Just under half (45.3%) of binge drinkers had not also used marijuana or tobacco.

**Table 3:** **Co-occurrence of additional substance use behaviours for those who smoked marijuana, smoked tobacco, or engaged in binge drinking in the past month**

	**n**	**Weighted % (95% CI)**
Smoked marijuana		
Only smoked marijuana	47	17.6 (11.5-23.7)
Also smoked tobacco but did not binge drink	42	14.2 (10.1-18.2)
Also engaged in binge drinking but did not smoke tobacco	60	21.4 (15.8-27.0)
Also both smoked tobacco and engaged in binge drinking	131	46.8 (40.1-53.5)
Smoked tobacco		
Only smoked tobacco	77	23.9 (18.9-28.9)
Also smoked marijuana but did not binge drink	42	13.0 (9.4-16.6)
Also engaged in binge drinking but did not smoke marijuana	58	20.2 (15.5-25.0)
Also both smoked marijuana and engaged in binge drinking	131	42.9 (36.9-48.9)
Engaged in binge drinking		
Only engaged in binge drinking	214	45.3 (39.9-50.6)
Also smoked marijuana but did not smoke tobacco	60	13.0 (9.7-16.3)
Also smoked tobacco but did not smoke marijuana	58	13.4 (10.2-16.5)
Also both smoked marijuana and tobacco	131	28.4 (23.0-33.7)

### Frequency of substance use behaviour

The association between comorbidity and substance use behaviour frequency is presented in the ordinal regression models in Table 4. Considering the adjusted odds ratios, the results suggest that number of substance use behaviours engaged in is a significant predictor of behaviour frequency. In each instance, respondents who reported a greater number of substance use behaviours also reported undertaking each more frequently.

**Table 4:** **Adjusted odds ratios in the ordinal logistic regression models for frequency of substance use by number of behaviours**

	**AOR (95% CI)**	***p*** **-value**
Marijuana^i^		
1 (only marijuana)	1	
2	1.66 (.82-3.39)	0.160
3	3.19 (1.66-6.13)	0.001
Tobacco^ii^		
1 (only tobacco)	1	
2	2.18 (1.32-3.60)	0.003
3	5.53 (3.15-9.74)	<0.001
Binge drinking^iii^		
1 (only binge drinking)	1	
2	3.58 (2.18-5.87)	<0.001
3	7.01 (4.42-11.10)	<0.001

^i^F(2,142) = 7.13, p < .01.

^ii^F(2,142) = 18.17, p < .001.

^iii^F(2,142) = 35.16, p < .001.

### Profile of substance use comorbidity

The influence of socio-demographic characteristics on degree of comorbidity is presented in the ordinal logistic regression model in Table 5. Considering the adjusted odds ratios for the selected predictor variables, the results suggest that gender, ethnicity, school decile status, past week income, social connectedness, and parental monitoring of income, monitoring of whereabouts and enforcement of rules are all significant predictors of an increasing degree of substance use comorbidity.

**Table 5:** **Adjusted odds ratios in the ordinal logistic regression model for socio-demographic characteristics, by degree of substance use comorbidity**

	**AOR (95% CI)**	***p*** **-value**
Gender		
Female	1.31 (1.03-1.68)	0.031
Male	1	
Ethnicity		
Māori	2.24 (1.72-2.92)	<0.001
Non-Māori	1	
School decile status		
Low	1.98 (1.38-2.84)	<0.001
Mid	1.27 (0.89-1.80)	0.181
High	1	
Past week income		
More than $30	2.42 (1.84-3.19)	<0.001
$16 to $30	1.87 (1.42-2.46)	<0.001
$1 to $15	1.07 (0.79-1.45)	0.677
None	1	
Social connectedness		
Low	2.15 (1.63-2.84)	<0.001
Medium	1.35 (1.04-1.76)	0.024
High	1	
Parental monitoring of expenditure		
Disagree or don’t know	2.37 (1.90-2.96)	<0.001
Agree	1	
Parental monitoring of whereabouts		
Disagree or don’t know	2.13 (1.67-2.71)	<0.001
Agree	1	
Parental rule enforcement		
Disagree or don’t know	1.37 (1.09-1.71)	0.006
Agree	1	

Model: F(12,132) = 33.79, p < .001.

Females were more likely to report a greater number of substance use behaviours than males. Māori ethnicity, low school decile status, higher weekly income and low social connectedness were also associated with an increasing degree of substance use comorbidity. Greater agreements that parents monitor expenditure, monitor whereabouts and enforce rules were associated with a decreasing degree of substance use comorbidity.

## Discussion

This is the first New Zealand study to provide data on the comorbid use of marijuana, tobacco, and binge drinking in a nationally representative sample of adolescents. The results confirm that a significant number of Year 10 students engage in substance use behaviour, with around one-in-five smoking marijuana, smoking tobacco, and/or binge drinking in the past month. Comorbid substance use was relatively common among those engaging in substance use behaviours, with around half of those engaging in one also engaging in another, and a core group of around 5% engaging in all three. The comorbid relationship between marijuana and tobacco was particularly strong, with nearly six-in-ten tobacco smokers also smoking marijuana. Further, those who engaged in a greater number of the substance use behaviours were likely to undertake each more frequently and therefore be at greater risk of subsequent associated harm; a finding consistent with international research [3,11,12]. The study also found common socio-demographic characteristics among those adolescents engaging in comorbid use of marijuana, tobacco, and binge drinking. Comorbid substance users were likely to be female, of Māori ethnicity, attend a low decile school, report a high past week income, have low social connectedness, and have parents who do not monitor their expenditure, monitor their whereabouts or enforce rules. These common factors are consistent with previous research [16,19-21] and support the contention that there is a common underlying vulnerability to substance use.

Given the high likelihood that adolescents engaging in one substance use behaviour will also be engaging in others, the literature recommends that public health interventions consider substance use as a whole, and that in order to effectively reach those at greatest risk interventions need to screen for, consider, and address the multiple links between substances [7,8,13,19,20,29]. However, this recommendation is only partially supported by the findings of the current study. While only a small proportion of substance-using adolescents used only tobacco or only marijuana, around half engaged in binge drinking without also using another substance, although those individuals who were binge drinking more frequently were more likely to also use the other substances. Therefore, these findings suggest that interventions targeting marijuana and tobacco use should indeed address the links with other substances, but it is possible that only those interventions targeting frequent binge drinking (as opposed to occasional binge drinking) will also benefit from doing so.

The findings also suggest that the audience of tobacco-only smoking adolescents likely to be reached by traditional tobacco control messages focusing solely on tobacco is small. The tobacco-only group represented less than three percent of the total Year 10 population, whereas just over eight percent smoked tobacco as well as marijuana and/or engaged in binge drinking. Therefore, tobacco control messages addressing multiple substances would have around three times a larger audience. Further, whereas a decade ago one-in-five Year 10 students in New Zealand reported smoking tobacco [5], tobacco control efforts over the years have reduced the rate found of smoking in the current study to a level nearly that of marijuana use (around one-in-ten) – and as observed here they have significant overlap. This raises the question as to whether further tobacco control efforts considering the marketing of tobacco can achieve efficient gains, assuming marijuana prevalence is a fair indicator of the base rate for use of a substance that is not commercially marketed. New Zealand tobacco control efforts may now have reached all but a core group. It may be difficult to further significantly reduce adolescent tobacco use when rates of comorbid substance use are so (comparatively) high unless tobacco control messages are widened to address multiple substances. For example, young people have reported difficulties in quitting smoking tobacco while still smoking marijuana [30] or consuming alcohol [31], so messages addressing these difficulties may be more useful.

It appears that binge drinking is relatively common by age 14 and 15, with almost one-in-five reporting binge drinking in the past month. The minimum legal purchase age of alcohol in New Zealand is 18 years, so the availability of alcohol to younger adolescents is clearly a problem. Comorbid substance use was less likely for binge drinkers compared with tobacco or marijuana users, indicating that comorbidity varies with substance accessibility and binge drinkers are less likely to be deviant than tobacco or marijuana users. Consequently, there is likely to be a larger audience for alcohol-specific interventions. Policy interventions targeting the social supply of alcohol to minors may therefore prove effective.

There may also be further risk factors for comorbid substance use that were not available in this study. In contrast to a previous study of a New Zealand birth cohort which found that Māori ethnic identification was no longer a risk factor for marijuana use when other factors were taken into account [32], Māori ethnicity did remain a significant predictor of substance use comorbidity in the current study. One key risk factor that has previously been found to relate to substance use, but was not collected in the current study, is parental substance use [33,34]. Future studies could include a wider range of risk factors to help build a more comprehensive understanding of adolescent comorbid substance use.

Our results derive from a conservative measure of substance use (that is, ‘past month’ compared to lifetime or past six month use), and with a younger age group (14 to 15 years old) compared to other measures in the adolescent substance use literature [9,10,20,35]. This suggests that a core group engaged in comorbid substance use behaviours is formed relatively early, in mid-adolescence, and highlights the need to intervene against substance use in this age group.

However, although the study has established the presence of comorbid substance use among 14 to 15-year-olds, the cross-sectional nature of the data means that the trajectory of substance use cannot be determined. Comorbid substance use appears constrained within a subgroup which shares those factors indicating vulnerability to substance use. However, it is possible that identified comorbid substance use at 14 and 15 years of age is an indicator of more widespread uptake of substance use in later adolescence. This aligns with Fergusson and Horwood’s [32] finding that more than two-thirds of a New Zealand birth cohort had used marijuana by age 21 years. On this interpretation, this study has identified an early-onset group of comorbid substance users. Indeed, previous research indicates that likelihood of comorbid substance use increases with age [8,35], so the size of this substance-using group could increase as the students grow older. Longitudinal research considering comorbid substance use is needed to determine which theory is supported. Interventions will be implied by such research results. If future research confirms psychological problems or social settings that impact on underlying vulnerability to comorbid substance use, then these could be targeted for intervention within the at-risk group. Alternatively, if comorbid substance use is a relatively common part of adolescent development, then it can be addressed at a broader or national level targeting experimentation with substance use in general.

There are two other limitations of the study that are also important to note. First, the study did not ask about the relatively common practice of mixing tobacco in with marijuana [36]. This may have implications for how marijuana or tobacco use are defined, as a previous study found that some marijuana users who self-identified as ‘non-smokers’ had smoked tobacco in the past month, and appeared to be perceiving themselves in terms of their use of marijuana without considering their tobacco use [37]. This issue has also been raised as a limitation in previous research [20], and future studies would benefit from making this distinction. Second, respondents were asked in a class room setting about illegal behaviours, and thus may have had concerns about confidentiality. However, to minimise response bias, the questionnaire was anonymous and respondents were advised that participation was voluntary.

## Conclusions

This study identifies the prevalence of comorbid use of marijuana, tobacco and binge drinking in 14 to 15-year-olds. Comorbid substance use is related to a greater frequency of engaging in substance use behaviour, highlighting a greater risk of subsequent harm arising from comorbid substance use compared with the use of one substance in isolation. Comorbid use is predicted by factors such as gender, ethnicity, school decile status, past week income, social connectedness, and monitoring and rule enforcement by parents. This supports the view that there is a common underlying vulnerability to substance use in certain individuals, and implies the need for specific interventions. Alternatively, this study may reveal an early indication of more common comorbid substance use in later adolescence. Further research is needed for clarification. What is clear, however, is that interventions targeting multiple substances are required – especially concerning dual marijuana and tobacco use.

## Endnotes

^a^The dollar amounts refer to New Zealand dollars (NZD). NZD$30 is equivalent to approximately USD$22 or GBP£15 as at February 2015.

^b^Being respectful of the potential limitations of an ordinal logistic regression model, multinomial logistic regression models were run concurrently and produced consistent results to those reported here.
